# Active Inference: Applicability to Different Types of Social Organization Explained through Reference to Industrial Engineering and Quality Management

**DOI:** 10.3390/e23020198

**Published:** 2021-02-05

**Authors:** Stephen Fox

**Affiliations:** VTT Technical Research Centre of Finland, VTT, FI-02044 Espoo, Finland; stephen.fox@vtt.fi; Tel.: +358-40-747-8801

**Keywords:** active inference, artificial intelligence, industrial engineering, information-theoretic entropy, Markov blankets, organization, prediction error, quality management, social, statistical process control, thermodynamic entropy, variational free energy

## Abstract

Active inference is a physics of life process theory of perception, action and learning that is applicable to natural and artificial agents. In this paper, active inference theory is related to different types of practice in social organization. Here, the term social organization is used to clarify that this paper does not encompass organization in biological systems. Rather, the paper addresses active inference in social organization that utilizes industrial engineering, quality management, and artificial intelligence alongside human intelligence. Social organization referred to in this paper can be in private companies, public institutions, other for-profit or not-for-profit organizations, and any combination of them. The relevance of active inference theory is explained in terms of variational free energy, prediction errors, generative models, and Markov blankets. Active inference theory is most relevant to the social organization of work that is highly repetitive. By contrast, there are more challenges involved in applying active inference theory for social organization of less repetitive endeavors such as one-of-a-kind projects. These challenges need to be addressed in order for active inference to provide a unifying framework for different types of social organization employing human and artificial intelligence.

## 1. Introduction

Active inference is a physics of life process theory of perception, action and learning. Active inference generates predictions. Active inference predictions are based on knowledge learnt from past situations and perceptions of present situations. Active inference minimizes errors between actions that are planned through the inference of predictions and what happens when the planned actions are taken. This is because prediction errors cause unwanted surprises, which individually or accumulatively can threaten survival by going beyond the limited number of states in which survival is possible. By contrast, minimizing prediction errors facilitates survival through least action [1,2]. Active inference is a corollary of the free-energy principle (FEP) which formalizes cognition of the autopoietic organization of living systems [3,4]. Within FEP, active systems must occupy a limited repertoire of states. This requires minimizing the long-term average of surprise associated with sensory exchanges with the world. Minimizing surprise enables them to resist a natural tendency to disorder. Surprise rests on predictions about sensations, which depend on an internal generative model of the world. In particular, although surprise cannot be measured directly, a free-energy bound on surprise can be, suggesting that agents minimize free energy by changing their predictions about what sensory inputs will come from actions or by changing the predicted sensory inputs through changing action [5].

The purpose of this paper is to relate active inference to social organization in order to explain opportunities for, and challenges in, active inference theory providing a unifying framework for social organization employing human and artificial intelligence. In particular, this involves relating important constructs in active inference to practice concerned with minimizing unwanted surprises. Limiting unwanted surprises from prediction errors underlies industrial engineering and quality management. Industrial engineering involves applying methods, such as task analysis and job design, in order to predict results, evaluate results, and improve results from processes during their development [6]. Quality management systems (QMS) involve documenting processes, which have been developed through industrial engineering, as process specifications, work procedures, etc.; monitoring processes for conformance to specifications etc.; and using observations of nonconformances to inform the further development of processes [7]. Both are focused on the continuous improvement of processes. The application of industrial engineering has been progressing around the world since the 1900s and quality management since the 1950s [8,9,10].

The paper is intended to contribute to bridging the gap between theoretical papers in active inference theory, which have been reported to be too opaque to be understood widely [11,12], and potential application opportunities in multi-intelligence social organization—that being social organization that employs human intelligence and different types of artificial intelligence [13]. Throughout the paper, theory-based practical examples are discussed that can provide common descriptions for scientists researching active inference and engineers who are interested in implementing active inference research in multi-intelligence social organization. They can provide widely applicable practical examples, because industrial engineering and quality management are applied in social organization in many different sectors around the world [14,15]. In order to be useful for the widest possible range of scientists and engineers, theory-based practical examples are provided of everyday social organization. Although previous papers by others have encompassed active inference up to the levels of sociocultural cognition [16] this is the first paper to address active inference in everyday organizational practice including industrial engineering and quality management.

The paper proceeds in eight further sections. In Section 2, Section 3, Section 4, Section 5 and Section 6, important constructs in active inference theory are related to social organization through reference to industrial engineering and quality management. Accordingly, readers who are conversant with active inference theory and have knowledge of industrial engineering and quality management could skip these sections. In Section 2, variational free energy is described in terms of the monitoring of social organization processes through statistical process control. In Section 3, prediction errors are described in terms of nonconformances in social organization processes. In Section 4, quality manuals are described in terms of generative models. In Section 5, Markov blankets are described in terms of boundary states in social organization. In Section 6, social organization survival is related to important constructs in active inference theory. In Section 7, it is explained that while artificial intelligence (AI) founded upon active inference theory can be useful for individual applications [17,18], active inference theory can also provide a unifying framework for the development and operation of social organization employing humans and many different types of AI. In Section 8, challenges in applying active inference theory to different types of social organization are explained. In Section 9, the paper’s principal contribution is stated and directions for future research are proposed.

## 2. Variational Free Energy

Within active inference theory, prediction errors leading to unwanted surprises are considered in terms of variational free energy (VFE). This is a technical term applied within complex statistical models including FEP models. VFE is a mathematical construct. VFE is a measure of how much variation there is between predicted sensory data and subsequent sensory data. VFE increases as the difference increases between what is predicted to happen and what does happen. At the same time, evidence for predictions decreases as difference increases between what is predicted to happen and what does happen. In other words, surprise and evidence are inverse functions, because surprisal is minimized when evidence for predictions is maximized. Hence, maximizing evidence lower bound is equivalent to minimizing surprise upper bound.

**Figure 1:**
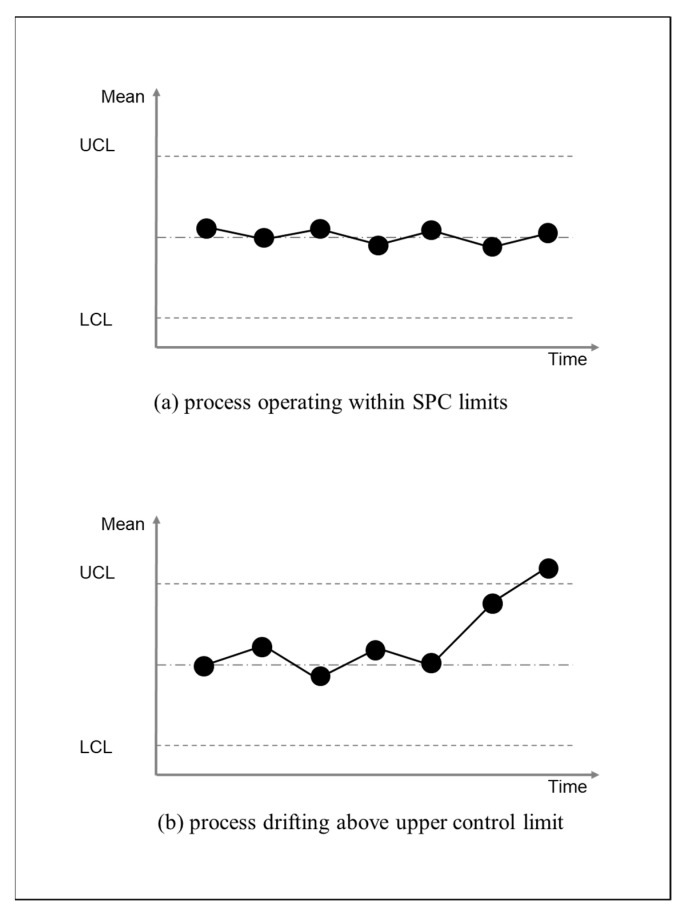
Statistical Process Control (SPC) chart of a process (**a**) operating within SPC limits but then (**b**) drifting beyond upper control limit (UCL).

In order to survive, humans need to live within existential limits by minimizing prediction errors [19,20]. In particular, minimizing prediction errors prevents human systems’ states from dispersing in a statistical sense by keeping the values of certain key variables within certain existential bounds: for example, some prediction errors can push humans outside of existential limits with catastrophic consequences. An example would be a person’s body temperature falling below the lower survival limit for body temperature, when the person has underestimated the amount of clothing needing to be worn when working outside in extreme cold at a remote location. Conversely, a person’s body temperature could go above the upper survival limit for body temperature if the person overestimates the amount of time that can be spent working outside in extreme heat at a remote location. As summarized in Figure 1, existential limits can be compared to the lower control limit (LCL) and upper control limits (UCL) of statistical process control (SPC) charts. In quality management, SPC charts are used monitor the conformance of processes to specifications, which are developed through industrial engineering [7,9]. As shown in Figure 1 below, SPC charts have lower control limits (LCL) and upper control limits (UCL). Processes need to remain within these limits (Figure 1a). Processes that go outside of these limits will not meet specifications that define intended states (Figure 1b).

SPC charts can be applied to any process and SPC limits can be related to survival limits: for example, there are control limits for processes for machining materials into parts. Any part machined beyond the process control limits will be thrown into the scrap bin. Similarly, existential limits can be related to a company’s survival in business: for example, a company will not survive if it underestimates the minimum time required to complete customer orders or conversely if it overestimates the maximum time required to complete customer orders. This is because underestimating can lead to winning lots of orders but making a financial loss for every order. Conversely, overestimating will lead to not winning orders. In either case, the company cannot survive.

Within active inference theory, predictions are precision weighted based on prior expectations of sensory data and empirical variance of sensory data [20]. Similarly, monitoring of predictions by SPC is precision-weighted based on prior expectations of sensory data: for example, predictions for machining materials into parts have levels of precision that are based on prior expectations of what needs to be monitored, e.g., width but not height; what level of machining precision is needed, e.g., millimeters not centimeters; and what level of empirical variance is inherent in the machining monitoring technology, e.g., fractions of millimeters. Similarly, order completion time can be measured in necessary levels of precision for management accounting and what level of empirical variance is inherent in the completion time reporting. Processes are developed during industrial engineering so that empirical variance is not more than three standard deviations below (i.e., LCL) or above (i.e., UCL) the process mean. If SPC indicates that a process is drifting towards a control limit, the process will be stopped.

There are some differences between measurements in SPC and VFE in FEP: for example, existential limits in SPC are always not more than three standard deviations below (i.e., LCL) or above (i.e., UCL) the process mean. Furthermore, SPC limits are defined explicitly in terms of process-specific quantities such as millimeters or hours. By contrast, VFE is not defined as mean, standard deviations, LCL and UCL in terms of process-specific quantities. Rather, VFE limits are implicit prior preferences in embodied cognition for sensory inputs. Nonetheless, both SPC and FEP are concerned with minimizing unwanted surprises that could undermine survival; and measurements in SPC and VFE in FEP quantify variation between predicted sensory data and subsequent sensory data.

## 3. Prediction Errors

Between SPC limits, social organization can survive a very wide variety of prediction errors with more than minimum VFE: for example, a company underestimating or overestimating for one contract will not put it out of business, provided the company learns not to repeat the prediction error. Indeed, social organization can only survive in the long-term if there is sufficient space between existential limits to allow for exploration [21,22]. However, just as with individuals, social organization needs to remain in nonequilibrium steady-states (NESS) by restriction to a limited number of states. This is because social organization involves living systems that need to resist the second law of thermodynamics in order to persist as bounded self-organizing systems over time. The restriction of states can be at the level of individual processes, such as the machining of materials into components within lower limit—LCL and upper limit—UCL expressed in fractions of millimeters. At the same time, restriction of states can be at the level of customer orders: for example, a company needs enough orders to remain in business (lower limit—LCL) but not more orders that it can fulfil (upper limit—LCL). Whatever the level, there will be differences between predicted sensory inputs and actual sensory inputs: i.e., there will be variational free energy, but these differences and arising variational free energy must be within survivable limits.

The more frequent are prediction errors—the more thermodynamic energy will be consumed in disorder than invested in productive work. Consider, for example, a person who intends to use an electric saw to cut a piece of wood. The person predicts sensory visual data will come from seeing the sawcut causing the one piece of wood to become two shorter pieces of wood of exactly the correct dimensions. However, there will be high VFE, for example, when the electric saw does not cut the wood accurately and scratches the person’s hand. This is because rather than the predicted sensory visual data, there is the unwanted surprise of the electric saw causing incoming sensory pain data as it scratches the person’s hand.

This would be followed by situation-specific instances of additional nonproductive energy consumption, which takes place in the context of the fundamental requirement to minimize the amount of thermodynamic energy needed to survive. In particular, the person using the electric saw needs to take remedial actions to gain information about the state of the hand, and take additional actions to care for the hand. The person also needs to dispose of the miscut piece of wood, acquire another piece of suitable wood, and do the cut again. At the same time, the person needs to take corrective actions that involve gaining information about why the electric saw did not cut right-first-time, and changing the process so that the electric saw does cut right-first-time in the future. This could be corrective action such as changing the saw guard.

Taking additional actions to address prediction errors comes at the cost of consuming more energy that in turn necessitates acquiring more energy through restorative actions: for example, the person needs to acquire more food because of extra actions arising from prediction errors. So, s/he needs to walk to a shop to buy another two sandwiches: one extra sandwich to address the depletion arising from prediction errors plus one more extra sandwich to address depletion from walking to the shop. The more remedial actions, corrective actions, and restorative actions have to be carried out, the more likely it is that a person and/or a company will be overwhelmed by what can be described as firefighting: in other words, becoming trapped in a quagmire of errors, deadline pressure, overtime working and energy depletion [23]. As illustrated in Figure 1 through reference to SPC, this can lead to drifting beyond of the existential limits for survival.

In order to prevent this, prediction errors are reported in QMS as nonconformances. These reports are analyzed by QMS management review. Whenever QMS management review identifies the reoccurrence of similar nonconformances, the underlying causes will be investigated and improved processes will be developed through the application of techniques such as the “Five Whys”. This is an iterative analysis technique that is applied to determine the root cause of nonconformances by repeating the question “Why?” with each answer forming the basis of the next question. The name “Five Whys” is based on observation that five is the number of iterations needed to resolve nonconformances [24].

**Figure 2:**
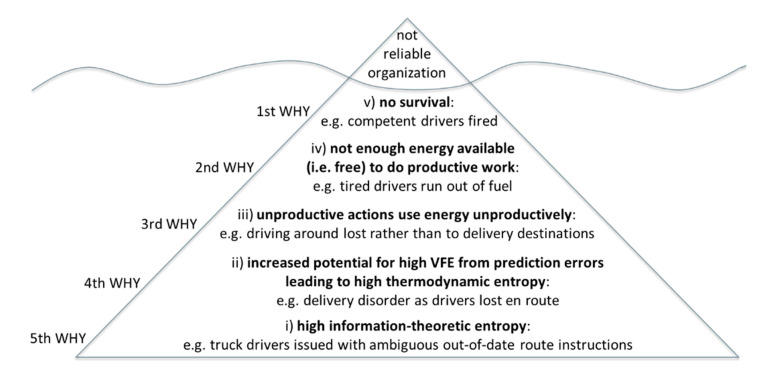
Five Whys shown as iceberg diagram reveals causes of unreliability.

As shown in the Five Whys iceberg diagram in Figure 2, VFE can be related to information-theoretic entropy, least action, and survival: for example, (i) high information-theoretic entropy in work instructions (ii) increases potential for high variational free energy (VFE) from prediction errors, which in turn increases thermodynamic entropy of (iii) unproductive actions that (iv) consume energy unproductively and (v) reduce thermodynamic free energy available for doing productive work that is necessary for survival. This could be (i) providing a truck driver with ambiguous out-of-date route instructions (ii) increasing the potential for getting lost and (iii) driving around unproductively and (iv) so not having enough fuel remaining to (v) complete the deliveries that are necessary to retain employment as a truck driver. As summarized in Figure 2, this can lead to the company not being reliable in its deliveries because the company is terminating the employment of truck drivers who are competent, but who are not issued with unambiguous up-to-date route instructions.

An iceberg diagram, such as Figure 2, is not a precise scientific representation. Rather, it is a illustrative representation of issues to be addressed in the improvement of social organization processes. Within this depiction, issues are arranged hierarchically in the order in which they are revealed by asking why. However, process improvement is focused on the underlying cause of process nonconformance. In this case, the cause is revealed by the fifth asking of why: it is that the instructions issued to truck drivers need to be improved to reduce information-theoretic entropy.

In order to reduce information-theoretic entropy, industrial engineering techniques, such as task analysis, failure modes and effects analysis, and job design can be applied to any aspect of work including delivering materials and cutting materials. Such techniques have the aim of enabling right-first-time process by anybody anywhere (i.e., minimal thermodynamic entropy process from minimal information-theoretic entropy process). In other words, foolproof processes. QMS nonconformance reports and QMS management review are applied to identify processes in need of improvement through further application of industrial engineering techniques.

Interrelationships between variational free energy (VFE), information-theoretic entropy, thermodynamic entropy and thermodynamic free energy is a topic of ongoing research by others [25,26]: for example, it has been argued that the energetics of active inference can be considered in terms of the probability of a particle having followed a given trajectory. In particular, it is argued that the amount of heat dissipated along a given trajectory is an expression of how surprising it would be to observe a system following the same trajectory backwards relative to forwards. Such research is at an early stage of development. In the future, it may be directly applicable to organizational endeavors involving industrial engineering and quality management.

Meanwhile, interrelationships between the mathematical constructs of variational free energy (VFE), information-theoretic entropy, thermodynamic entropy and thermodynamic free energy can described in words that are less precise but that can provide an overview. In particular, VFE increases as the difference increases between what is predicted to happen if actions are taken and what does happen when those actions are taken. The more prediction errors there are, the more thermodynamic energy will be consumed amidst the thermodynamic entropy of remedial actions, corrective actions, restorative actions, and firefighting. This will leave less thermodynamic energy available for doing productive work comprising productive actions. Thus, if VFE is high, then thermodynamic entropy (e.g., energy not available for doing productive work) will be high and thermodynamic free energy (e.g., energy available for doing productive work) will be low. Hence, variational free energy (VFE) can have an inverse relationship to thermodynamic free energy. This is because the bigger a prediction error, which can be considered in terms of bigger VFE, the smaller can be the thermodynamic free energy available to do productive work. Here, it is important to note that this overview is relevant to social organization that has a limited supply of thermodynamic energy: for example, it is relevant to any social organization that needs to keep away from the thermodynamic entropy of remedial actions, corrective actions, etc., in order to have the revenue from satisfied customers to employ personnel and to pay its energy bills. For social organization that is heavily subsidized this overview is not applicable because there is not a limited amount of energy available to such social organization. Rather, additional energy will be supplied in the hope of doing productive work no matter how much energy is lost amidst the disorder of remedial actions, corrective actions, etc.

## 4. Generative Models

Within active inference theory, embodied cognition implements generative models comprising beliefs about the world, in particular, generative models that generate predictions about how sensory inputs are caused by things outside the brain. Whatever beliefs are about, beliefs are Bayesian beliefs: that is, beliefs that are quantified subjectively as probabilities, for example, a belief that a welding process operates correctly 99 times out of 100 operations. However, generative models can be inaccurate due to false beliefs about how sensory inputs are caused by things outside of the brain. Such generative models may be described as fictive [27], and lead to what has been described as wishful seeing where people see what they already believe [28,29]: for example, a production manager who started work as an apprentice doing welding tasks, who enjoyed doing welding tasks, and has friends who still do welding tasks could have overly positive beliefs about welding processes. This could lead to seeing a belief that welding processes operate correctly 99 times out of 100 operations when welding processes are operating less than 90 times out of 100 operations. Wishful seeing and other delusions are not restricted to a few neural diversity outliers. Rather, they are commonplace in human endeavors. Moreover, they can lead to huge prediction errors [30,31].

Quality management systems (QMS) seek to reduce such negative effects of subjective beliefs by documenting processes which have been developed through industrial engineering, as process specifications, work procedures, etc.; monitoring processes for conformance to specifications etc.; and using observations of nonconformances to inform the further development of processes [7]. Work procedures etc., are recorded in the QMS’s quality manual. In particular, process specifications, work procedures, etc., can be described as being beliefs that are based on knowledge from past situations: those being the past situations that occurred during industrial engineering, such as digital simulations and physical tests. They are beliefs that generate predictions that can be described as expected posterior probabilities given prior probabilities. Prior probabilities are what did happen at the conclusion of the development of the process during industrial engineering: e.g., process operated correctly (i.e., right-first-time) at least 99 times out of 100. Expected posterior probabilities are what is expected to happen in operation of a process: e.g., expected to operate correctly at least 99 times out of 100. A process can be described as being a capable process [7] when it operated correctly at least 99 times out of 100 at the conclusion of industrial engineering (i.e., prior probability). That process can be described as being under control [7] when it does operate correctly at least 99 times out of 100 when it is in operation (i.e., actual posterior probability).

In relation to FEP, expected posterior probability can be considered in terms of expected free energy (EFE). In other words, EFE is the expected difference between the sensory inputs that are predicted to come from a planned process (i.e., preferred sensory inputs) and sensory inputs that do come from that process when it is carried out (i.e., actual sensory inputs). As summarized in Figure 1, from the point of view of industrial engineering and quality management, the difference between preferred sensory inputs and actual sensory inputs should be not more than the quantity between three standard deviations below (LCL) or above (UCL) the process mean: i.e., should be within six sigma [32,33,34]. Within QMS, only prior probabilities of at least 99 times correct operation out of 100 are an acceptable basis for beginning the operation of a process, and only actual posterior probabilities of at least 99 times out of 100 are an acceptable basis for continuing the operation of a process. Thus, the aim is to minimize process risk to less than one incorrect operation in 100 operations of the process [35]. It is necessary to minimize process risk to less than one incorrect operation in 100 operations because otherwise there will be need for remedial action, corrective action, etc., in more than one in 100 operations of a process. As discussed above, social organization that is not subsidized cannot survive high levels of remedial actions, corrective actions, etc.

**Figure 3:**
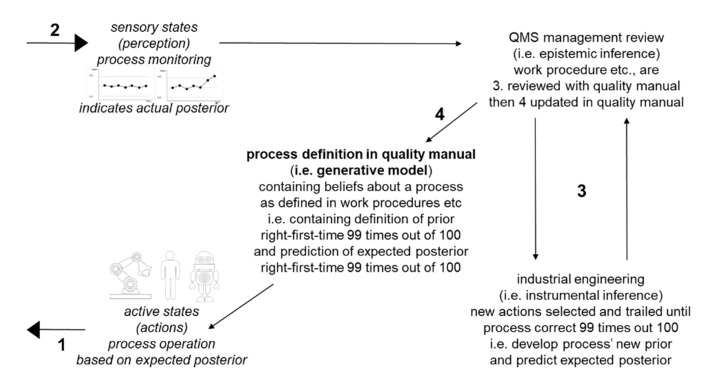
Quality management system (QMS) quality manual as generative model comprising beliefs about processes. 1. Process operated based on expected posterior of right-first-time operation 99 times out of 100. 2. Process monitoring indicates actual posterior is worse than right-first-time 99 times out of 100. 3. QMS management review initiates further industrial engineering until prior is right-first-time 99 times out of 100 and expected posterior can be predicted to be right-first-time 99 times out of 100. 4. Process definition (i.e., beliefs about process) updated in quality manual (i.e., generative model).

Prediction errors occur when a process is not carried out right-first-time in at least 99 percent of cases. This can be because the process is not under control or is not capable. The process may not be under control because of special causes, such as the usual source of materials supply is temporarily not available and lower quality materials have to be used for a few months. Alternatively, the process is inherently not capable because it has an intrinsic flaw that was not identified during industrial engineering due to the limited number of physical trials that could be carried out: for example, tool wear could be more than expected, which causes reduced machining accuracy and hence conformance to process specification in only 50 out 100 operations of the process. In other words, actual posterior probability is 50 out of 100 operations rather than the 99 out of 100 operations, which was preferred, predicted and expected. Hence, there are remedial actions, corrective actions, etc., in about half of cases. As summarized in Figure 3, when the monitoring of processes through QMS methods such as statistical process control reveals that a process does not operate correctly at least 99 times out of 100, the process has to be improved. This can encompass addressing both previously overlooked intrinsic flaws in process capability and new causes of disruption to process control. First, patterns are identified in nonconformance reports. Then, techniques such as the Five Whys are applied to identify root causes. After that, the process is improved through the application of techniques such as job design in further industrial engineering.

As summarized in Figure 3, process improvement can involve instrumental inference in selecting new actions to be included in the process during industrial engineering and epistemic inference in updating the definition of the process in the QMS quality manual through QMS management review. Within active inference theory, instrumental inference is a subset of active inference in which actions serve primarily to regulate causes of perceptual variables [20]. Epistemic inference is a subset of active inference to enhance generative models so as to enable enhanced prediction error minimization in the long run [20]. After trials in industrial engineering demonstrate sufficient evidence that the process’ expected posterior probability has been improved e.g., from right-first-time in 50 out 100 operations to right-first-time in at least 99 out of 100 operations, the process can go back into operation. Monitoring of the process will resume when the process is back in operation. Again, variance of empirical data should not be more than three standard deviations below or above the mean.

Exactly how beliefs are updated in embodied cognition is a topic of ongoing research, which involves specialist mathematics to describe relationships between prior probabilities and posterior probabilities [36,37]. By contrast, updating beliefs through active inference in industrial engineering and quality management involves applying well-established practical methods. In particular, planning what should happen by active inference involves developing processes through applying industrial engineering techniques. The resulting processes are documented as process specifications, work procedures, etc., within quality management system (QMS) quality manual. In terms of active inference, these are beliefs originating in industrial engineering about what will happen in future processes. They are beliefs that processes will be carried out right-first-time in at least 99 percent of cases. This is because industrial engineering and quality management aim to develop and to operate right-first-time processes irrespective of location and personnel. Hence, the capability of the most successful social organization to operate with high conformance to process specifications, work procedures, etc. in many different countries and do so with high productivity [38,39].

In terms of exploration for information and exploitation for reward, industrial engineering involves iterations of exploration carried out to reduce ambiguity in the operation of processes: for example, a rule in the industrial engineering method Design for Assembly is to make parts either symmetrical or clearly asymmetrical. This is done so that it is immediately and unequivocally apparent how a part is to be put into a subassembly. In other words, it is done so there is no ambiguity about how the part is picked, orientated and placed either by a human or by a robot [40,41]. Subsequently, it is intended to profit from the costs invested in industrial engineering explorations. In particular, it is intended to profit from the rewards of exploiting processes that are defined in QMS quality manual to operate right-first-time at least 99 times out of 100: in other words, it is intended by organizations that they will obtain rewards from processes that have a very low risk of not operating right-first-time.

## 5. Markov Blankets

Within active inference theory, it is argued that systems comprise hierarchical layers of nested boundaries: for example, the boundaries of cells within trees, the boundaries of trees within forest patches, and boundaries of forest patches within grasslands. It is argued that bounded systems engage in active inference based on the separation of internal states and external states. This is consistent with the separation of internal states and external states being a fundamental necessity to resist the second law of thermodynamics in order to persist as bounded self-organizing systems over time [42]. Furthermore, it is argued that boundaries comprise states that separate internal states and external states [43]. This is consistent with, for example, the boundaries of forest patches amidst grasslands having their own states as transition zones between them [44]. Here, it is important to note that edge effects can emerge from such ecological boundaries. These are effects of increased variety, which can emerge spontaneously [45].

Within active inference theory, a technical term used to describe separation of internal states and external states is Markov blankets [46]. This term is related to partially observable Markov decision process (POMDP), which is named after the Russian mathematician Andrey Markov. POMDP is a mathematical framework for modeling decision making in situations where the outcomes are partly under the control of an agent and partly random; and in which the agent cannot directly observe everything. Instead, the agent must maintain a probability distribution over the set of possible states: for example, humans do not directly observe external states. Rather, humans cope with a vast barrage of incoming light patterns by extracting features from them [47]. A Markov blanket is a set of variables through which internal states and external states interact. Furthermore, a Markov blanket itself can encompass many sensory states and active states: for example, the skin can be described as a Markov blanket for the human body [48], and the skin is a sensor packed with nerves that has many active states [49].

Markov blankets are ascribed to real-world systems in order to model the conditional independence between internal states and external states [50,51,52]: for example, conditional independence between a production process and the environment in which it is carried out. Markov blankets are not real features of real-world systems. Rather they are intuitive post hoc ascriptions made in order to model real-world systems [5]. Hence, any ascription of a Markov blanket is subjective and can be changed over time as the modeler’s focus changes and/or as real-world systems change in the opinion of the modeler [53]. Thus, as summarized by phrases such as: the map is not the territory, Markov blankets are typical of subjective ascriptions made in the modeling of systems [54]. More specifically, it has been argued in relation to Markov blankets and other important constructs in active inference theory that the math is not the territory [5].

For species, boundaries may evolve naturally over millennia and boundaries may then be unchanging for further millennia. In comparison, social organization may need to define boundaries in weeks and be ready to change them in days [55,56]. The definition of social organization boundaries in quality management involves active inference about social organization boundaries. Prediction errors about social organizational boundaries can threaten social organization survival. This can be prediction error about which environment to try to survive in: for example, trying to do business in a market segment that has many entrenched competitors could lead to too few orders. By contrast, trying to do business in a market segment that has no competitors could lead to too many orders.

In other cases, many processes may fit very well within a market, but survival can be threatened when only one process does not. Consider, for example, do-it-yourself (DIY) furniture assembly processes compared to the DIY furniture installation processes.

Furniture kits include product-specific components, component-specific connectors, and connector-specific tools. These have come from iterations of industrial engineering carried out to minimize assembly risk. In particular, to ensure that there is only one way to assemble products such as cupboards and wardrobes. Although furniture companies may not be considering their industrial engineering in terms of minimizing VFE, it is apparent that its aim is to minimize errors between what it intends to people to see when looking at its furniture kits and what people do see when looking at its furniture kits: for example, see that the connector-specific tool is for the component-specific connectors for the product-specific components. Moreover, the introduction of furniture kits can eliminate many former sources of VFE, which could arise from trying to make furniture oneself: for example, VFE arising from waiting in vain for wood to be delivered by a company that does not provide its truck drivers with up-to-date unambiguous route instructions. Plus, VFE arising from cutting wood and inadvertently hand when trying to make furniture components oneself.

However, the installation of furniture assembled from kits is more likely to lead to VFE from prediction errors than the assembly of furniture from kits. This is because prediction errors can arise from extraneous causes such as installation being carried out in situations with details that cannot be envisaged fully by the furniture company. In other words, although there may be little risk in furniture assembly, this can be followed by much ambiguity in furniture installation. For example, industrial engineering has been carried out to develop fixing packs containing fixing components and fixing instruction pictures that do not include written text that could be lost in translation. However, predetermining possible prediction errors in fixing assembled furniture to walls in millions of different buildings around the world is very challenging.

With regard to state boundaries in fixing an assembled cupboard to a wall, the internal state can be the embodied prior knowledge. In particular, the embodied prior knowledge about fixing furniture to walls of the person who will fix the cupboard to the wall. The external state can be the room with the wall to which the cupboard will be fixed. The probability of the internal state being compatible with the task can be at least 99 times in 100 attempts, because the fixing pack has been developed to be foolproof: i.e., anybody anywhere can use it. The probability of the external state being compatible with the task can also be 99 times in 100 attempts, because the fixing pack has been developed to be suitable for any type of wall.

**Figure 4:**
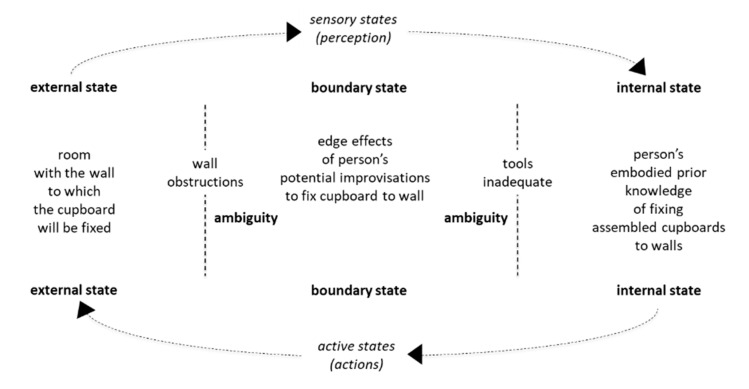
Example of ambiguity within boundary state.

However, as summarized in Figure 4 below, there can be ambiguity in the boundary state. The borders of the boundary state are the borders of the internal state and the external state. At the border of the internal state are the tools that the person will use to do the fixing work. At border of the external state are physical objects that are close to the wall that the cupboard will be fixed to. Within the boundary state, there can be edge effects, i.e., spontaneous emergence of variety.

Here, it is important to note that the fixing pack provided by the company cannot include fixing tools such as stepladder, electric drill, screwdriver, etc: for example, if the person does not have exactly the correct type of screwdriver to fix the fixing screw into the wall, the person may not be able to turn the fixing screw all the way into the final position. Then, the person might hammer the screw into the final fixing position, which leads to a weak fixing. This is an example of ambiguity emerging from the internal border of the boundary.

Also, there can be ambiguity emerging from the external border of the boundary state: for example, the room may be full of crockery, pots and pans taken out of old cupboards that are being replaced. As a consequence, the wall may be obstructed and the person may not be able to get enough force behind the screw driver to turn the screw into its final fixing position. This could also lead to the person hammering the screw into final fixing position, which leads to a weak fixing. Alternatively, hammering the screw could arise from both borders when there is not the correct type of screw driver and it is not possible to get into a good position to apply force to the screw driver. Whether the edge effects emerge from the internal border or the external border or both, the weak cupboard fixing may eventually come loose when the cupboard is loaded, for example, with crockery, pots and pans.

## 6. Survival

A summary of active inference constructs in social organization practice is provided in Table 1 below. As summarized in Figure 1 VFE upper bound is represented by the maximum quantity between SPC limits in industrial engineering and quality management. Furthermore, the precision weighting of predictions is present in industrial engineering and quality management. As summarized in Figure 2, application of process improvement techniques, such as the Five Whys, reveals interrelationships between variational free energy (VFE), information-theoretic entropy, thermodynamic entropy, thermodynamic free energy, action, and survival. In doing so, they reveal why it is imperative to minimize prediction errors.

As summarized in Figure 3, industrial engineering techniques are applied iteratively until there is sufficient evidence (i.e., priors) that processes can be introduced into operations without prediction errors (i.e., no differences between priors and posteriors). Thus, industrial engineering techniques are applied until expected posteriors match priors. QMS monitoring for nonconformances, i.e., for prediction errors, leads to any nonconforming processes being improved through further industrial engineering until there is sufficient evidence that their operation will not lead to further nonconformances. Then, the improved processes can be reintroduced into operations. Thus, iterations of industrial engineering generate predictions that are increasingly compatible with survival through least action by eliminating nonproductive actions.

Within active inference theory, states are described in terms as Markov blankets, and expected free energy (EFE) is defined as risk plus ambiguity [57]. Here, ambiguity can be added to inherent process risk when social organization cannot bring boundary states under control between predictable internal states and predictable external states. The boundaries of social organization’s internal states in relation to external states are defined and monitored by quality management. Accordingly, as summarized in Figure 4, if ambiguity revealed by quality management monitoring cannot be eliminated through industrial engineering, then boundaries should be shifted to eliminate threats to social organizational survival. In terms of active inference theory, this is can be compared to niche construction whereby organisms modify their environment to steer their own evolutionary trajectory through their regulatory mechanisms and actions [58].

Overall, the definition of social organization boundaries defined in QMS should allow for exploration as well as exploitation. This is as important for the survival of human organizations as it is for any other form of life [59,60]. The need for social organizations to be able to combine exploitation and exploration is summed up in the term leagile. This is term is a portmanteau of the words lean and agile where lean maximizes efficiency in meeting stable demand while agile maximizes efficiency in meeting changing demand [61]. For example, many social organizations have to be able to explore how to survive in markets that change through new competitive forces. Hence, industrial engineering and quality management are not confined to a few well-established types of work. Rather, they can be applied to new work arising from new market demands. Also, industrial engineering and quality management are applicable to innovative mobile factories that can take processes anywhere as they are to traditional fixed factories [62].

## 7. Active Inference as a Unifying Framework

**Figure 5:**
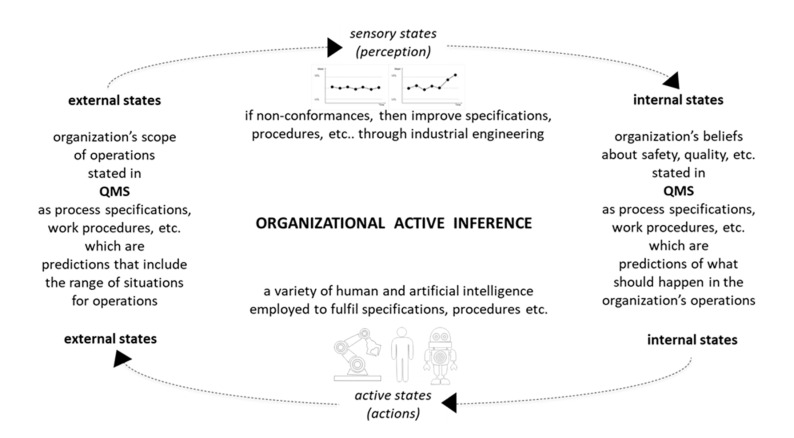
Unifying framework for multiintelligence social organization.

Computational active inference can be useful for individual applications [17,18]. However, many multi-intelligence social organizations inevitably comprise a wide range of different computer systems, which have different ontological bases as well as different computer languages: for example, even one mobile industrial robot can have a mobile platform from one developer/vendor and a robot arm from another developer/vendor: each with different software. Accordingly, an important application of active inference theory can be as a unifying social organization framework, which as summarized in Figure 5 is operated through quality management systems (QMS), via interoperability protocols at an upper level of multitier enterprise architecture.

In the following paragraphs, using production work as an example, eight advantages are described for active inference as a unifying framework for multi-intelligence social organization.

For example, physical production is dependent upon embodied natural intelligence and artificial intelligence among individuals and groups [63,64,65], which carry out physical work in order to survive in markets. Apropos, active inference provides a framework for embodied perception, action and learning, which can be applied to natural life and to artificial life [66]: for example, to evolutionary robotics [67].

Production also depends upon embodied action, and action is inherent in active inference [68]. In particular, active inference encompasses epistemic inference, which involves reducing prediction errors by updating beliefs through sensory inputs from active observation of current situations, and instrumental inference that involves reducing prediction errors by selecting actions to change current situations [69]. For example, commercially-available mobile industrial robot (MIR) platforms have sufficiently advanced narrow AI to move around a factory and learn while doing so [70]. MIR can make predictions of the shortest route for the delivery of materials in a factory. Then, they can take the action of following the shortest route prediction. Subsequently, if they encounter an obstacle, they can update their factory map and make a new prediction, and so on. Thus, they carry out rudimentary active inference.

In addition, active inference encompasses a wide scope of embodiment from the interoception of individuals [20] to the sociocultural learning of groups [16]. Notably, active inference theory research investigates effects of emotions on cognition [71]. Here, it is important to note that artificial intelligence, as well as human intelligence, encompasses emotion [72].

Furthermore, active inference is a comprehensive physics of life description [73], which encompasses relationships between entropy, action and energy. This is important because multi-intelligence production organizations need to be able to minimize these in order to survive. This can be simplified by active inference focusing on minimizing prediction errors, which in turn minimizes entropy, action and energy.

Importantly, active inference replaces the traditional reward function of reinforcement learning with the ability to learn beliefs about what should be done in situations through the reduction of prediction errors. Thus, active inference’s basis in beliefs takes perception, action and learning away from always prioritizing immediate rewards [74]. This is important because always prioritizing immediate rewards can undermine long-term survival. For example, dishonest signaling can offer immediate rewards, but in the longer-term undermines the integrity of the signaling system and the survival of the group [75]. Apropos, dishonest reporting can undermine reporting systems and lead to social organization collapse [76], which can undermine the survival of many individuals [77,78,79]. Moreover, the reduction of prediction errors through active inference accommodates exploitation, not just exploitation driven by immediate rewards [21].

Wherever explorations may lead, active inference can make human–AI interaction (HAII) more explainable because AI inference can have the same overall basis for inference as humans. That is active inference based on beliefs that are updated through iterations of the active observation of situations and/or actions in situations. Crucially, the definition of beliefs can align the actions of humans and robots in multi-intelligence in production work [80].

In doing so, active inference can facilitate survival with total least action overall. For example, production short-cuts can offer immediate least action but can cause accidents, which lead to more total action due to having to deal with the accident in addition to having to do the work. Production short-cuts can also undermine production quality, which can lead to remedial actions, corrective action, and restorative actions. Accordingly, active inference can facilitate total least action for survival when safety beliefs and quality beliefs are defined as sources of intrinsic motivation [57,81] that can override task-specific short-cuts, which are otherwise appealing due to time pressures, worker fatigue, etc., [82].

Overall, active inference being a unifying principle for multi-intelligence social organization can make HAII more productive. This is because human interactions with robots can be facilitated by taking into account internal contexts such as beliefs, external contexts such as situations, and how they can affect each other [83,84,85].

## 8. Challenges in Applying Active Inference Theory to Different Types of Social Organization

### 8.1. Markov Blankets

As explained in Section 5, Markov blankets are not real features of real-world systems. Rather they are intuitive post-hoc ascriptions made in order to model real-world systems. However, the ascription of Markov blankets to real-world social organization can be challenging.

Consider, for example, the challenge of ascribing Markov blankets in nested hierarchies to project organizations compared to mass production organizations [86]. In the mass production of consumer goods, there are well-defined hierarchies of predefined components as follows: part, subassembly, assembly, product. Furthermore, there are well-defined hierarchies of social organization as follows: tier 3 supplier, tier 2 supplier, tier 1 supplier, and original equipment manufacturer (OEM). Tier 3 suppliers, such as small contract manufacturing companies, are at the bottom and OEMs, such as Electrolux or Toyota, are at the top.

By contrast, during the project production of one-of-a-kind capital goods such as headquarters buildings, social organization is in matrices within which they are in a functional organization, such as an architectural firm or roofing company, while at the same time as working on several different projects at several different physical locations. There are also matrix structures from the sociocultural point-of-view. For example, a financial department may have sociocultural affiliation to the accountancy profession and to the particular economic sector, e.g., construction sector, as well as to the company. Furthermore, parts, subassemblies, assemblies and products are not predefined in project production of one-of-a-kind capital goods. Rather, definition is only finished when the one-of-a-kind capital good is complete. Subsequently, that definition is not reused because each one-of-a-kind capital good is intended to be unique.

Hence, as reported in the extensive literature about interface problems in project production, there is persistent cultural and physical ambiguity about the delineation of which social organization is responsible for what production [87]. For example, when do physical components become the legal responsibility of a site production company instead of the legal responsibility of the component production company? Is it when the components are delivered to the production site; is it when the components are incorporated into the product as it is being produced; is it when the components are paid for? In mass production, such questions are settled in advance through the engineering of supply chains. In project production, by contrast, there is often not enough time to settle all such questions in advance, and different companies can have contradictory procurement conditions that contribute to legal disputes [88]. Hence, the ascription of Markov blankets could be a recurring challenge throughout project production, which may only be resolved by reference to decisions made in courts of law long after the project is completed.

### 8.2. Generative Models

As explained in Section 4, embodied cognition implements generative models comprising beliefs about the world. Social organization is concerned with aspects of the world that will affect the operation of its processes. Accordingly, social organization will try to predefine those aspects of the world. In terms of active inference theory, this is can be compared to niche construction whereby organisms modify their environment to steer their own evolutionary trajectory [58].

In some cases, social organization involves highly repetitive operation of fully predefined processes in an entirely controlled environment: for example, the mass production of consumer products, such as white goods or family cars, in a factory. Such social organization practices are engineered to be “external-state-proof”. In other words, they are engineered so that whatever happens outside of the social organization, mass production will not be disturbed. For example, task analysis and job design are applied in order to enable anybody from anywhere to carry out work right-first-time or so that production can be fully automated. Hence, even if there is a shortage of personnel due to changes in external states, production can be carried out by people who can come from anywhere and can have no previous experience. Furthermore, mass production supply chains are engineered for resilience to external changes [89].

By contrast, in other cases, social organization involves the operation of a few predefined processes in conjunction with many processes that are not fully predefined in new environments: for example, project production of capital goods, such as headquarters buildings, at a new construction site [86]. Efforts are made to make project production “external-state-proof”: for example, through the prefabrication of building elements inside factories [90]. However, much of project production has to be situated within external states that change from one project to the next to such an extent that it is not possible to make all processes “external-state-proof”: for example, when external states include regulatory authorities and component suppliers that may never have been encountered before.

Many project production organizations do operate quality management systems (QMS), but their work procedures have to accommodate a wide range of possible processes carried out at a wide range of possible locations. For example, project production companies will carry out the definition of project-specific critical tasks and noncritical tasks together with the predicted minimum and maximum amount of time for each in accordance with QMS. However, they do so with very incomplete information [91]. Charts have milestones, which mark the start and the end of phases of project work in relation to time in the project schedule. Milestones can be considered as being points in time where local minima of uncertainty can be established [25]: for example, milestones provide checkpoints where planned budget and time (i.e., priors) can be compared to current best estimates of expenditure and duration (i.e., preferred posteriors). From these local minima of uncertainty, new predictions of cost and time can be made, which can be based on the creation of modifications to both path and destination [92]. Although cost and time can be far more than predicted at the outset or at previous milestones, all parties can be too deep into the project to quit [93].

The formulation of project charts could give the impression that getting to a project destination involves navigating from milestone to milestone. However, in terms of the explore/exploit dynamic [21], project production involves much more exploration than exploitation. Hence, more wayfinding than navigation is involved. In particular, wayfinding involves the ability to create novel routes, which are based on understanding a wider frame of reference than navigating along a preset route [94]. Wayfinding involves creating novel routes through changing situations by making nonconscious reference to subjective prior knowledge and conscious subjective reference to current situations and semantic information [95,96].

Overall, it is important to differentiate between social organization that is involved in exploration during industrial engineering followed by recovery of the costs of exploration through exploitation monitored by QMS (e.g., mass production), and other social organization that is involved in continual exploration that is monitored by QMS but with limited potential for process improvement (e.g., project production). Apropos, it is important to distinguish between social organization that is involved in navigating and social organization that is involved in wayfinding. Consider, for example, escalating commitment to a failing course of action in project production. It has been argued that there are four recurring stages [93]. From the outset, it is argued that the project destination will be perfect. This encourages delusional and/or deceitful descriptions of the path to the destination [97]. In turn, this encourages escalation of commitment to sticking to the path to the destination, while scapegoating the weakest project participants with accusations of their making the path difficult. Subsequently, future-perfect envisaging of the project destination and path to destination misrepresentation is redoubled until eventually the less-than-perfect project destination is reached at cost and time that far exceeds those predicted at the outset [98]. Efforts have been made for decades to improve the formulation of project charts, for example by reducing overly optimistic underestimates of cost and time [99,100]. Nonetheless, cost and time overruns continue: sometimes spectacularly in projects such as Berlin Brandenburg Airport, which went four billion euros over budget cost and eight years over scheduled time [101]. Thus, despite QMS, generative models in many projects continue to be unproductively fictive.

### 8.3. Prediction Errors

In mass production, prediction errors are reduced through industrial engineering to make internal states “external-state-proof”. In such social organization, subjective priors, subjective perceptions of current situations and subjective perceptions of semantic information can be engineered out of operations to such an extent that anybody from anywhere can quickly start productive work. Consider, for example, that people without any previous experience in catering work can begin productive work in a global fast-food chain’s restaurant after only a few minutes of on-the-job training. By contrast, the practices of project production involve more interactions between subjective prior knowledge, subjective perceptions of current situations and subjective perceptions of semantic information. Such interactions are the scope of pragmatics [102,103,104].

Although it is has been argued in 2020 that FEP may be a formal theory of semantics [105], pragmatics is inherent within inference in any situation that is not fully predefined and standardized for all possible participants in advance. Consider, for example, that within the active inference literature, it is argued that sensory inputs from perception are not prescreened for relevance to a situation, but instead they are rendered relevant when they lead to the selection of an action or to the updating of a generative model [25]. Similarly, within the pragmatics literature, in particular within Relevance Theory, it is argued that information is deemed relevant when it yields a positive cognitive effect, such as improving knowledge on a certain topic [104].

As summarized in Table 2, there can be many reasons why pragmatics subsumes semantics and leads to prediction errors: for example, inept priors and inept preferred posteriors can come from lock-ins, path dependencies, and success traps, which place emphasis on past experiences over new information. They can also come from management fads and technology hype that driven are driven by social contagion. All of these involve groupthink [106]. This may be because humans evolved, when hunter gathers, to place emphasis on knowledge considered most important for survival [107] and individuals typically seek to survive through inclusion within groups [108,109]. Hence, individuals may seek to survive by learning from filtered information that supports the preconceptions of groups, which they believe can support their individual survival [110,111] rather than seek information that supports the efficient reduction of prediction errors in external states.

Meanwhile, interoception, exteroception and proprioception can be affected by many internal and external factors, such as emotional fatigue from long working hours, poor eyesight from bad lighting and densified fascia from cramped working spaces [112]. Furthermore, active inference will often have to be carried out amidst the bounded rationality of imperfect information and limited time [113] while muddling through from one situation to another [114]. Action can also be based on habit [115,116], rather than on deliberative reflective thinking that involves more effort [117,118,119]. In addition, active inference may be concerned with satisficing rather than optimizing. In other words, actions being satisfactory to internal states and assumed sufficient for continued survival of internal states: rather than optimizing external states [120]. Satisficing can be influenced by an unconscious preference for least cognitive effort [121] and subconscious preference for least social resistance [122]. For example, optimization of external states may be served best by learning based on rigorous analyses of new technologies, but satisficing may be served best by going along with superficial technology hype [106].

Such examples of pragmatics can lead to many prediction errors that lead to many remedial actions, corrective actions, restorative actions, and firefighting. Accordingly, it is far from sufficient for active inference to encompass semantics. Rather, pragmatics need to be addressed by the application of active inference to any social organization that cannot engineer out all influence of subjective perceptions of semantic information, subjective perceptions of current situations, and subjective priors. Notably, pragmatics has more influence than semantics in wayfinding [123,124,125,126].

### 8.4. Variational Free Energy

As stated in Section 3 above, social organization need to remain in nonequilibrium steady-states (NESS) by restricting themselves to a limited number of internal states. This is because social organization involves living systems that need to resist the second law of thermodynamics in order to persist as bounded self-organizing systems over time. As stated in Section 5, self-organizing systems persist in internal states that are bounded from external states by Markov blankets. Active inference should be an account of autopoiesis in dynamic terms, provided that social organization is (i) at nonequilibrium and (ii) can be differentiated from its their environment. NESS means that a social organization persists over a nontrivial timescale and does not dissipate, for example, through its internal resources being scattered into an external state by being taken into the possession of external creditors via bankruptcy proceedings. Differentiation brings conditional dependencies between internal states and external states with corresponding information geometries based on internal states that have Bayesian beliefs about external states. In particular, the internal state parameterizes a probability distribution over the external states, which represents the causes of sensory inputs from external states. In this way, the NESS density towards which the social organization evolves in terms of its intrinsic information geometry can be interpreted as a generative model of extrinsic information geometry. This generative model can be considered as a joint probability density over internal states and external states. When there is no prediction error in the internal state’s parameterization of probability distribution over the external states, internal gradient flow on variational free-energy (VFE) over time can be equivalent to external gradient flow on VFE. At its simplest, the minimization of surprise can be regarded as a gradient descent [127,128].

For example, a company may predict that its internal costs caused by fulfilling ten thousand external orders will be nine million euros, plus the company needs to satisfy its shareholders by there being one million euros profit from fulfilling those external orders. Meanwhile, the company will predict that ten million euros will come from external orders. If there is a prediction error, actual sensory inputs will not match actual sensory inputs. In particular, the company will not see nine million euros going out and ten million euros coming in. A large prediction error could lead to the company dissipating, for example, through its internal resources being scattered into an external state by being taken into the possession of its unpaid suppliers. Alternatively, the company could be acquired and merged into one its large unpaid suppliers that seeks to move up the value chain from tier 1 to OEM. In the case of acquisition and merger, the ascription of Markov blankets would need to be changed in legal documents. As illustrated by this example, cash flows have some potential to be a focus of VFE modelling. Another example is that, project production companies that have too many orders to process may increase the profit margin on the quotations that they submit in response to external enquiries. This is based on the belief that increasing quoted prices will reduce the number of external orders.

For interactions between humans and different types of artificial intelligence during the processing of orders, VFE modelling could be focused on number of actions possible within the goal of least action to achieve order completion in accordance with QMS. For example, taking short-cuts could lead to least action in the short-term but not in the long-term. This could be because taking short-cuts that lead to least action in the short-term can lead to nonconformances and accidents that have to be addressed through nonproductive remedial actions, corrective actions, etc. Nonconformances and accidents can also damage reputation, which can lead to the loss of external orders that leads to the social organization not surviving. Consider, for example, the manufacture, assembly and fixing of cupboards within control limits. Companies operate within control limits because companies that operate the production of cupboards outside of control limits cannot survive. Rather, they will expire amidst the high thermodynamic entropy of unproductive energy consumption within the disorder of remedial actions and corrective actions. Accordingly, cupboard companies seek to define control limits and minimize prediction errors within them through continuous development of their processes by industrial engineering and QMS monitoring. In doing so, cupboard companies minimize variational free energy in the active inference of the humans who will use cupboard kits. This is because humans who open cupboard kit boxes see what they expect to see: cupboard-specific components, component-specific connectors, and connector-specific tools, which their actions assemble in accordance with visual instructions. Thus, through industrial engineering informed by market research and QMS monitoring there can be a continuous cycle of active inference within which a company seeks to determine individuals’ expectations for sensory inputs and then meet individuals’ expectations for sensory inputs [129,130,131]. Such repetitive cycles in the development and monitoring of processes can facilitate the quantification of VFE. By contrast, the possible quantification of VFE is hindered when companies are involved in project-specific cycles of trying to determine individuals’ expectations for sensory inputs and then trying to meet individuals’ expectations for sensory inputs with change from one project to the next.

## 9. Conclusions

### 9.1. Principal Contribution

The principal contribution of this paper is to relate active inference theory to everyday practice in social organization. Active inference is a corollary of the free-energy principle (FEP), which formalizes cognition in the autopoietic organization of living systems that resist the second law of thermodynamics by occupying a limited repertoire of states, thus, persisting as bounded self-organizing systems over time. It is explained throughout this paper how industrial engineering and quality management better enable social organization to occupy a limited number of states in order to persist as bounded self-organizing systems over time rather than dissipating into their surrounding environment.

In Section 2, it is explained that statistical process control (SPC) involves social organization defining existential limits for processes. The distance between these limits can be considered in terms of the VFE upper bound. In other words, the maximum unwanted surprise in sensory inputs that can be survived. In Section 3, it is explained that unwanted surprise from prediction errors about processes can lead to nonproductive remedial actions, corrective actions, and restorative actions. From the perspective of thermodynamics, this involves more energy being lost in entropy and less energy being available for productive work. From the perspective of business, this involves incurring extra costs without generating income, which can lead to dissipation.: for example, through internal resources being scattered into the surrounding environment via bankruptcy proceedings brought by creditors. Accordingly, as explained in Section 4, social organization can monitor processes through QMS in order to identify prediction errors as soon as possible and to carry out further iterations of industrial engineering to address the sources of prediction errors. This involves QMS manuals being generative models that continually update beliefs about what actions will keep social organization within survivable limits. As explained in Section 5, in relation to Markov blankets, the positioning of boundaries has a determining influence over what sources of prediction errors can be addressed by industrial engineering and quality management. However, as explained in Section 6, social organization cannot survive in the long-term by staying within exactly the same boundaries and exploiting existing knowledge about how to minimize prediction errors within those boundaries. Rather, survival depends upon retaining capacity for exploration for new information when external environments change.

From this perspective, industrial engineering and quality management can be seen as being successful natural experiments in active inference that have taken place within diverse social organization in many different countries over many decades [14,15,132,133,134]. However, as explained in Section 8 and summarized in Table 3 below, industrial engineering, quality management, and active inference constructs are not readily applicable to all types of social organization.

### 9.2. Directions for Future Research

Active inference constructs are most readily applicable when social organization is involved in the high repetition of predefined processes. By contrast, there is much less potential for application of industrial engineering, quality management, and active inference constructs when social organization is involved in one-of-a-kind processes: for example, nested hierarchies of Markov blankets cannot be ascribed to shifting matrix structures that are common in one-of-a-kind projects. Hence, further refinement of active inference constructs is needed to enable wide application to social organization employing human and artificial intelligence. Apropos, the practical examples in the paper can provide starting points for research into the challenges of ascribing Markov blankets, defining generative models, handling pragmatics, and modelling VFE in real-world social organization.

Such future research should investigate where comprehensive mathematical modeling of social organization in terms active inference theory is feasible, viable, and beneficial. Where comprehensive mathematical modelling it is not possible and worthwhile, future research could investigate where active inference framing of social organization in terms of major active inference constructs can be beneficial. For example, as much of marketing is concerned with social organizations seeking to determine individuals’ expectations and then meeting individuals’ expectations, active inference theory could provide a new fundamental framing of marketing. Active inference theory could also provide a structure for the framing of carbon off-sets and other exchanges between internal states and new conceptualizations of external states. Another direction for future research could be to investigate the potential for active inference constructs to provide the bases of simple rules for social organization design and operation. Here, it is important to note that simple rules have proven to be highly successful in improving the performance of many different types of social organization [135,136,137].

Interrelated topics for future research could be as follows: social organization for making hospital building components; social organization for the construction of a hospital building; social organization for operation of a hospital; social organization for the operation of healthcare services including several hospitals. For each topic, preliminary research could seek to determine which approach is the most likely to be feasible, viable and beneficial: mathematical modelling or organization structuring or simple rules. Subsequent research could focus on the development of the appropriate approach for each topic. Research into interrelated topics could also investigate whether the application of simple rules during organizational design could facilitate structuring social organization in terms of active inference constructs, which in turn could better enable mathematical modelling for the improved operation of multi-intelligence organizations.

## Figures and Tables

**Table 1:** Active inference constructs in industrial engineering and quality management practice.

Construct	Practice
Variational free energy (VFE)	Difference between predicted sensory inputs and actual sensory inputs when monitoring processes e.g., SPC for machining of components
VFE upper bound	Maximum VFE within which survival is possible, e.g., maximum between the lower and upper control limits of SPC charts
Precision-weighting	What aspects of process are monitored with what level of precision within variance limits e.g., width of part machining in fractions of millimeters, which must always be within six standard deviations
Expected free energy (EFE)	Expected difference between preferred sensory inputs from process operation as defined in QMS process specifications, etc., and actual sensory inputs from process operation
Prior	Evidence from process trials during industrial engineering: prior probability needs to be right-first-time at least 99 times out of 100
Expected posterior	Expected right-first-time process operations in social organization practice defined as acceptable in QMS process specifications, work procedures, etc.: needs to be right-first-time at least 99 times out of 100
Actual posterior	Actual process operation in social organization practice: needs to be right-first-time at least 99 times out of 100
Preferred posterior	Within QMS, it is preferred that the actual posterior is the same as the expected posterior because then there will be no need for remedial actions, corrective actions, restorative actions, and additional industrial engineering
Prediction error	Difference between acceptable percentage right-first-time process and actual percentage as recorded in nonconformance reports
Epistemic inference	Updating definitions of process during process improvement based on analysis of nonconformance reports
Instrumental inference	Updating what actions will be included in the process during process improvement
Markov blanket	Boundary state between the internal state of the social organization and the external state of the environment when operating processes defined in QMS
Internal state	Social organization processes developed during industrial engineering for minimum risk in the process environment
External state	The environment, which is developed for minimum risk in relation to the process defined in QMS
Risk	Difference between what happens in social organization operations given what is planned to happen in QMS
Ambiguity	Arises in boundary state between social organization operations planned in QMS and the environment
Least action	Facilitated by positioning social organization boundaries to eliminate ambiguity and by minimizing risk within social organization boundaries
Survival	Facilitated by social organization least action within QMS that allows for exploration as well as exploitation

**Table 2:** Pragmatic sources of prediction errors.

Active Inference Phase	Source of Prediction Error		
	Top–Down	Bottom–Up	Intermediate
Perception	Availability of inept priors	Intero-, extero-proprioception affected by e.g., emotion	Bounded rationality
Action	Focused on inept preferred posteriors	Attention affected by low signal-to-noise ratio (S/N)	Habit
Learning	Updating of inept priors	Focus on sociocultural conformance	Satisficing

**Table 3:** Potential for applying existing active inference constructs to different types of social organization.

Active Inference Construct	Type of Social Organization	
	Repetitive Predefined Processes (e.g., Mass Production)	One-of-a-Kind Processes (e.g., Project Production)
Markov blankets	Nested hierarchies are applicable to organizational structures but not necessarily to sociocultural affiliations	Shifting matrices in organizational structures and sociocultural affiliations can limit application of nested hierarchies
Generative models	Generative models for exploration during industrial engineering followed by generative models for exploitation monitored by QMS are applicable	Generative models for continual exploration are applicable but need for research into potential for increased application of generative models for exploitation
Prediction errors	Prediction errors can be minimized by engineering out and monitoring for negative influence of subjective priors, subjective perceptions of current situations and subjective perceptions of semantic information	Pragmatics need to be addressed because prediction errors persist due to difficulty of engineering out all negative influence of subjective perceptions of semantic information, subjective perceptions of current situations, and subjective priors
VFE	Possible quantification of VFE facilitated by continuous cycles of market research and process improvement within which individuals’ expectations for sensory inputs are determined and then met through the engineering and monitoring of highly repetitive processes	Possible quantification of VFE hindered by project-specific cycles of trying to determine different individuals’ expectations for sensory inputs, which change from one project to the next, and then trying to meet those different individuals’ expectations for sensory inputs

## Data Availability

Not applicable.
